# Latency of Renal Calculus

**Published:** 1898-06-11

**Authors:** 


### LATENCY OF RENAL CALCULUS.

The somewhat important fact that renal calculus may remain quite latent, so far as symptoms are concerned, for considerable periods was well illustrated in a case recently reported by Mr. Charles A. Morton, surgeon to the Bristol General Hospital. The patient, a boy, was admitted with well-marked symptoms of renal calculus, but under the influence of rest in bed these completely subsided, so completely, in fact, that he could run about and jump downstairs without any pain, and there was no tenderness in the loin on firm pressure or sharp percussion. The same thing had occurred before, he having been in hospital nine months before for lumbar pain, and having been discharged apparently cured. In the interval there had been no pain until the attack for which he was readmitted. Yet, on examination by the X-rays, a shadow was seen lying over the twelfth rib, one and a quarter inch from the spine. An operation was performed, and on pressing the finger over the anterior surface of the kidney the stone could be felt, covered by only a thin layer of renal tissue. The stone on removal was found to measure three-quarters of an inch by half an inch, and on it was a process about one-third of an inch long covered with crystalline oxalates. The boy recovered completely. It is a matter of very great interest to observe how completely the symptoms passed away under the influence of rest. Perhaps the stone dropped back into its bed—the cavity which it exactly fitted—and thus gave rise to no trouble until it became displaced again; but, anyway, the case proves that complete remission of symptoms must not be taken too confidingly as meaning neuralgia of the kidney, or gout, or uricacidæmia, or any one of the fifty things to which men cling in the hope that they may not really have a calculus after all.

